# The NK1R-/- mouse phenotype suggests that small body size, with a sex- and diet-dependent excess in body mass and fat, are physical biomarkers for a human endophenotype with vulnerability to attention deficit hyperactivity disorder

**DOI:** 10.1177/0269881116658992

**Published:** 2016-07-26

**Authors:** Katharine Pillidge, David J Heal, S Clare Stanford

**Affiliations:** 1 Department of Neuroscience, Physiology and Pharmacology, University College London, London, UK; 2 RenaSci Ltd, Nottingham, UK

**Keywords:** Attention deficit hyperactivity disorder, body mass index (mBMI), body fat content, body length, NK1/TACR1 receptor, obesity

## Abstract

The abnormal behaviour of NK1R-/- mice (locomotor hyperactivity, inattentiveness and impulsivity in the 5-Choice Serial Reaction-Time Test) is arguably analogous to that of patients with attention deficit hyperactivity disorder (ADHD). Evidence suggests that small body size and increased body weight are risk factors for ADHD. Here, we compared the body size, body mass and body composition of male and female NK1R-/- mice and their wildtypes that had been fed either standard laboratory chow or a high-fat (45%: ‘Western’) diet. Male NK1R-/- mice from both cohorts were approximately 7% shorter than wildtypes. A similar trend was evident in females. Male NK1R-/- mice fed the normal diet weighed less than wildtypes but the ‘body mass index’ (‘mBMI’: weight (mg)/length (cm)^2^) of female NK1R-/- mice was higher than wildtypes. When given the high-fat diet, the mBMI of both male and female NK1R-/- mice was higher than wildtypes. There were no consistent genotype or sex differences in protein, ash or water content of mice from the two cohorts. However, the fat content of male NK1R-/- mice on the Western diet was considerably (35%) higher than wildtypes and resembled that of females from both genotypes. We conclude that a lack of functional NK1R is associated with small body size but increases vulnerability to an increase in mBMI and fat content, especially in males. This phenotype could also be evident in ADHD patients with polymorphism(s) of the *TACR1* gene (the human equivalent of *Nk1r*).

## Introduction

Our findings from a series of studies have led us to propose that abnormalities in the behaviour of mice with functional ablation of the NK1 (substance P-preferring) receptor gene, *Nk1r*, are analogous to those seen in attention deficit hyperactivity disorder (ADHD). This proposal is based on several lines of evidence.

First, comparisons of the locomotor behaviour of NK1R-/- mice and their wildtypes have revealed that the former genotype typically displays locomotor hyperactivity in a range of different experimental settings (Fisher et al., 2007; Herpfer et al., 2005; Moyes et al., 2016; Porter et al., 2015a, 2015b).

Secondly, in the 5-Choice Serial Reaction-Time Test (5CSRTT), NK1R-/- mice typically score more omission errors (an index of inattentiveness) and carry out a higher number of premature responses, which is an index of a form of (‘waiting’) impulsivity (e.g. Yan et al., 2011). By contrast, NK1R-/- mice did not express excessive ‘false alarms’ (another index of impulsive behaviour (response inhibition)) in the 5-Choice Continuous Performance Test (5C-CPT; Porter et al., 2016). However, these mice performed excessive perseverative responses in both the 5CSRTT and the 5C-CPT (Pillidge et al., 2016; Yan et al., 2011): this behaviour is thought to reflect the repeated cognitive ‘checking’ behaviour expressed by ADHD patients (Gürkan et al., 2015).

Thirdly, one, or more, of these behavioural abnormalities of NK1R-/- mice is ameliorated by all four compounds that are licensed for treatment of ADHD: guanfacine (inattentiveness: Pillidge et al., 2014a); atomoxetine (impulsivity (premature responses): Pillidge et al., 2014b); d-amphetamine and methylphenidate (locomotor hyperactivity and perseveration: Pillidge et al., 2016; Yan et al., 2009, 2011).

Finally, evidence for an association between polymorphism(s) of the *TACR1* gene in humans, which is equivalent to the *Nk1r* gene in mice, and ADHD has been replicated (e.g. Sharp et al., 2014; Yan et al., 2010). This is interesting in light of associations between *TACR1* with bipolar disorder (Sharp et al., 2014) and alcoholism (Blaine et al., 2013), both of which show prominent comorbidity with ADHD.

On the basis of all this evidence, we have proposed that humans with *TACR1* polymorphism(s) comprise an etiologically distinct subgroup of ADHD patients. However, when carrying out all these studies, we noted that NK1R-/- mice seemed smaller than their wildtypes. There are many reports of an association between small body size and ADHD (Ptacek et al., 2009; Spencer et al., 1996), which is evident even after exclusion of factors that are known to increase the risk of both ADHD and low birth weight (e.g. maternal smoking and alcohol misuse). In fact, evidence from one case-controlled study of probands suggested that, as an independent risk factor for ADHD, low birth weight could account for at least 14% of all cases (Mick et al., 2002). Somewhat paradoxically, ADHD is also associated with a high body mass index (BMI) (Erhart et al., 2012; Hubel et al., 2006; Waring and Lapane, 2008; but see Curtin et al., 2005) that qualifies as obesity (BMI ⩾ 30 for adults or ⩾ 95^th^ percentile for children) and which extends into adulthood (Cortese et al., 2013, 2016). This association seems bidirectional because a high incidence of ADHD in obese individuals is also evident (Agranat-Meged et al., 2005; Altfas, 2002; Fleming et al., 2005). Furthermore, older children with ADHD have a higher tendency to be overweight, despite being smaller, than children without ADHD (Hanć et al., 2015).

Prompted by these findings, we compared body size, body mass and body composition of NK1R-/- and wildtype mice with the aim of determining whether or not these genetically-altered mice express physical abnormalities (small body size and excess body weight) that are more common in ADHD patients than comparator groups and which are regarded as risk factors for ADHD. Also, in view of evidence for sex differences in comorbid obesity and ADHD (Byrd et al., 2013; Kim et al., 2011; Van Egmond-Frohlich et al., 2012) we carried out these measurements on both male and female mice of both genotypes.

## Materials and methods

All procedures complied with the Animals (Scientific Procedures) Act (UK) (2010/63/EU) and had received local ethical approval at University College London.

### Animals

All the mice were bred at University College London and housed in a room held at 21 ± 2°C, 45 ± 5% humidity, with a 12/12 h light/dark cycle (lighting increased in steps from 07.00 to 08.00 h and decreased in steps from 19.00 to 20.00 h). Food and water were freely available at all times. We studied inbred homozygous mice because these mice express all the diagnostic abnormalities seen in ADHD (locomotor hyperactivity, impulsive behaviour, inattentiveness and perseveration). Hyperactivity, inattentiveness and perseveration are also evident in the homozygous (F2) offspring of heterozygote (F1) parents, but their impulsive behaviour arises from an interaction between a lack of functional NK1R and an, as yet unidentified, factor in the breeding environment (Porter et al., 2015b).

The study used a total of 77 age-matched, weanling mice (Cohort 1: 40; Cohort 2: 37). Differences in the litter sizes account for the unequal sample sizes of the two cohorts. The two genotypes (NK1R-/- and their wildtypes) derived from the same background colony of 129/Sv × C57BL/6J mice that were crossed with outbred MF1 mice more than ten generations ago (De Felipe et al., 1998). To avoid prolonged isolation of the mice in individual cages, they were all group-housed as littermates (2–5 per cage). The home-cages incorporated environmental enrichment (cardboard tunnels and tissue for nesting material) and were cleaned twice weekly (bedding: 3Rs Bedding Pty, Ltd).

#### Study design

*Cohort 1* (normal diet): Male and female mice of the same genotype were randomly assigned to breeding pairs. Ten male and ten female pups of each genotype were weaned onto standard lab chow (18% of total calorific value derived from fat: 2018 global Rodent Diet, Harlan) at three weeks of age. At 6 weeks (±1 day) of age, their body length (nose to tail) was measured, under anaesthesia, before culling by cervical dislocation.

*Cohort 2* (high-fat, ‘Western’ diet): A second batch of mice, from different breeding pairs and/or their litters, were weaned onto high-fat diet (45% of total calorific value derived from fat: Research Diets, NJ, USA) at 3 weeks of age. Every animal and the food remaining in each cage were weighed daily. The amount of food consumed per cage each day was corrected for the total weight of the mice within. After 28 days (i.e. at 7 weeks of age) the body length of the mice was measured, before they were culled, as before (see above). In this cohort, ‘Cage’ was treated as the experimental unit for measurements of food consumption. Measurement of body length could not be carried out ‘blind’ because the two genotypes have a different coat colour.

All carcasses were frozen at −20°C for subsequent chemical analysis of their fat, ash, protein and water content.

### Chemical analysis

The procedures were based on those described by Eisen and Coffey (1990). In brief:

***Water*:** All the carcasses were reweighed and frozen at −80°C for at least 5 h before freeze-drying (Heto PL9000) for 2 weeks at a shelf temperature of −25°C. The dried carcasses were then reweighed, to enable calculation of the water content of the carcass, and then stored in sealed jars in drying cabinets. Immediately before the chemical analysis, the dried carcasses were milled (Buchi Mixer B-400 homogeniser) and the samples analysed, as described below.

***Ash*:** Approximately 1 g of each milled carcass was placed in a silica crucible and fired at 600°C for 6 h in a muffle ashing furnace (Carbolite, OAF 11/1) after which the cooled crucibles were reweighed. The residual ash content of each sample was weighed: the total ash content (g) of the original carcass was estimated, from the original carcass weight, by extrapolation from the weight of ash in the sample.

***Fat*:** Carcass fat content was determined by a modified Soxhlet extraction protocol. Samples of carcass (~1 g) were weighed into cellulose extraction thimbles (Whatman 26 mm × 60 mm: 2800-266) and plugged with approximately 0.5 g of cotton wool. Petroleum ether (90 mL, Fisher 40–60°C: P/1760/17) was used to extract the fat from each thimble using a Tecator Soxtec HT2 system (Foss, UK)/Tecator Soxtec 2050 system (Foss UK Ltd, Wheldrake, UK), with a modified manufacturer’s protocol (35 min extraction, 30 min wash and a 10 min drying period). After boiling the sample in the solvent, the fat was extracted by evaporation and the weight of the extracted fat used to calculate the total fat content (g) of the original dried sample. The data for one subject (a female NK1R-/- mouse in Cohort 1) was excluded from the data-set due to partial spillage of the sample during the extraction process.

***Protein*:** The protein content of the samples was calculated following measurement of their nitrogen content using the Kjeldahl assay. Using a Tecator 2012 (FOSS, UK) digestion block, approximately 0.3–0.4 g of each carcass sample was digested for 1 h at 420°C, in a mixture of 10 mL of concentrated sulphuric acid (Fisher S/9240/PB17), two Kjeltab CQ catalyst tablets (containing potassium sulphate and copper sulphate) and an antifoam S tablet (sodium sulphate and silicone antifoam, Thompson & Capper). A FOSS 2001 Scrubber Unit with sodium hydroxide solution and water was used to neutralize and remove any acidic waste gases. Subsequently, 40 mL of 10 M sodium hydroxide (Fisher J/7800/21) and 20 mL water were added to the cooled digested samples and steam was bubbled through individual samples using a Tecator 2020 distilling unit / FOSS 2200 Kjeltic Auto Distillation unit (FOSS, UK). Each sample was distilled into 30 mL of Kjeldahl receiver solution (4% boric acid with bromocresol green/methyl red indicator, Fisher K/0200/21). The addition of sodium hydroxide neutralized the acid and produced ammonia, which was ‘captured’ by distillation into the receiver solution. Each sample was then titrated with 0.1 M volumetric grade hydrochloric acid (Fisher J/4350/17): this enabled estimation of the NH^3+^ content of the solution, which is proportional to the sample protein (amino acid) content.

### Statistics

InVivoStat (version 3.2) was used for statistical analysis of the data (Bate and Clark, 2014; Clark et al., 2012). Data for Cohorts 1 and 2 were analysed separately. Diagnostic plots were constructed routinely to confirm normality of the raw data and homogeneity of sample variance. In only one case did appreciable deviation from normality make it necessary to transform the data-set (RANK transform (indicated in Results)) before proceeding with parametric analyses. Univariate analysis of variance (ANOVA) was used to analyse the overall effects of the main factors (Genotype and Sex), or their interaction, on body length, size and body composition. Pairs of data were compared using the *post-hoc* LSD test.

Differences in the time course for changes in body weight and food consumption for the two genotypes / sexes when fed the high-fat diet were analysed using repeated measures ANOVA, with ‘Genotype’ and ‘Sex*’* as between-subjects factors and ‘Day’ as a within-subjects factor. ‘Mouse’ and ‘Cage’ were treated as the experimental units when analysing data for changes in body weight and food consumption, respectively (see above). For statistical analyses of food consumption, the sample numbers ((wildtypes) males (3)/females (2) and (NK1R-/-) males (3)/females (3)) were adequate for comparison of the main factors (Genotype and Sex), but not for assessment of any interaction between them.

## Results

### Body length

**Figure 1.**
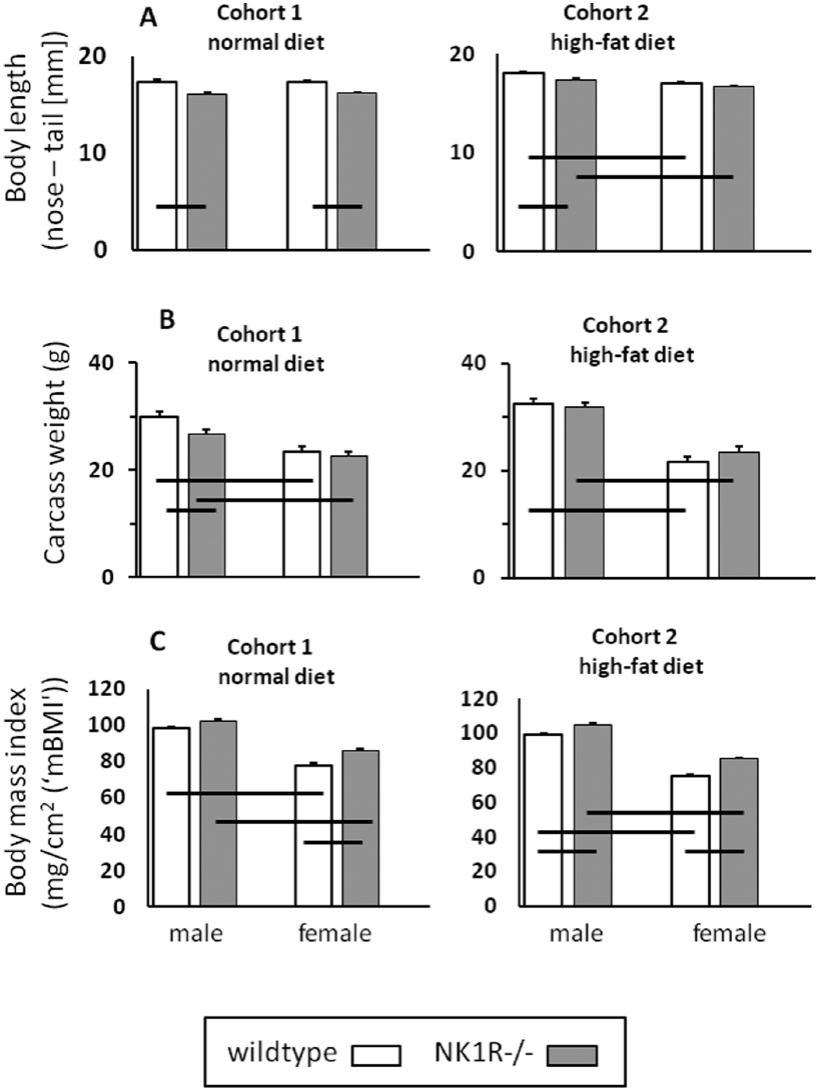
(A) Body length (nose to base of tail) (mm), (B) body weight (g) and (C) body mass index (mg/cm^2^) of male and female NK1R-/- and wildtype mice. Mice were weaned onto either standard laboratory chow (Cohort 1) or a high-fat (‘Western’) diet (Cohort 2). *N* = 8–10 per group. Data show mean ± SEM. Solid lines, linking two groups, indicate statistically significant differences of at least *p* < 0.05.

NK1R-/- mice from both cohorts were shorter, overall, than their wildtype counterparts (genotype: F_(1,37)_ = 46.62, *p* < 0.0001 (Cohort 1); F_(1,33)_ = 9.52, *p* = 0.004 (Cohort 2)]. Only female NK1R-/- mice in Cohort 2, given the high-fat diet, were not shorter than their wildtypes (Figure 1(A)).

### Body weight

NK1R-/- mice in Cohort 1 (i.e. fed the normal diet) weighed less, overall, than their wildtypes (genotype: F_(1,36)_ = 6.21, *p* = 0.02), especially the males (LSD: *p* < 0.01). However, neither male nor female NK1R-/- mice in Cohort 2 weighed less than their wildtypes (Figure 1(B)).

Both genotypes of male mice weighed more than their females in both cohorts (F_(1,36)_ = 46.38, *p* < 0.0001 (Cohort 1); F_(1,33)_ = 180.2, *p* < 0.0001 (Cohort 2)) (Figure 1(B)).

### Body mass index (‘mBMI’)

By analogy with the use of BMI in humans as an index of body density, we calculated the ratio of body mass:body length (nose–tail; cm)^2^ (‘mBMI’) for these mice. With the exception of males from Cohort 1, mBMI of NK1R-/- mice was higher than that of wildtypes (F_(1,36)_ = 10.34, *p* < 0.01 (Cohort 1); F_(1,33)_ = 20.6, *p* < 0.0001 (Cohort 2)) and the mBMI of males was consistently higher than that of females (F_(1,36)_ = 106.31, *p* < 0.001 (Cohort 1); F_(1,33)_ = 158.6, *p* < 0.001 (Cohort 2)] (Figure 1(C)).

### Changes in body weight and food eaten during 28 days of high-fat diet

**Figure 2.**
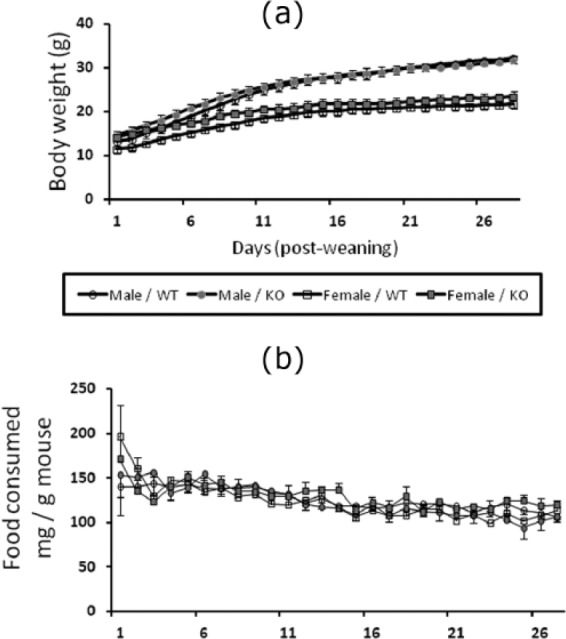
(a) Time course for weight gain of male and female NK1R-/- (KO) and wildtype (WT) mice fed the high-fat diet for 28 days. Male mice gained more weight than females, but there was no overall difference between the two genotypes for either sex. (b) There was no difference in the amount of food consumed after correction for the body weight of the mice (mg/g) by the two genotypes and two sexes over the 28 days.

There was no overall difference in the time course for the increase in body weight of the two genotypes which were fed the high-fat diet (Figure 2(a)). However, the body weight of males increased more quickly than the females, during 28 days of high-fat diet (sex*day: F_(1,27)_ = 98.4, *p* < 0.0001]. The progressive increase in the weight of the males also persisted throughout the 28 days of the study, whereas the weight of the females tended to stabilise after approximately 2 weeks.

Despite these genotype and sex differences, the amount of food consumed by the mice was not affected by either genotype or sex, when this variable was corrected for the body weight of the mice (Figure 2(b)).

### Fat (mg/g body weight)

**Figure 3.**
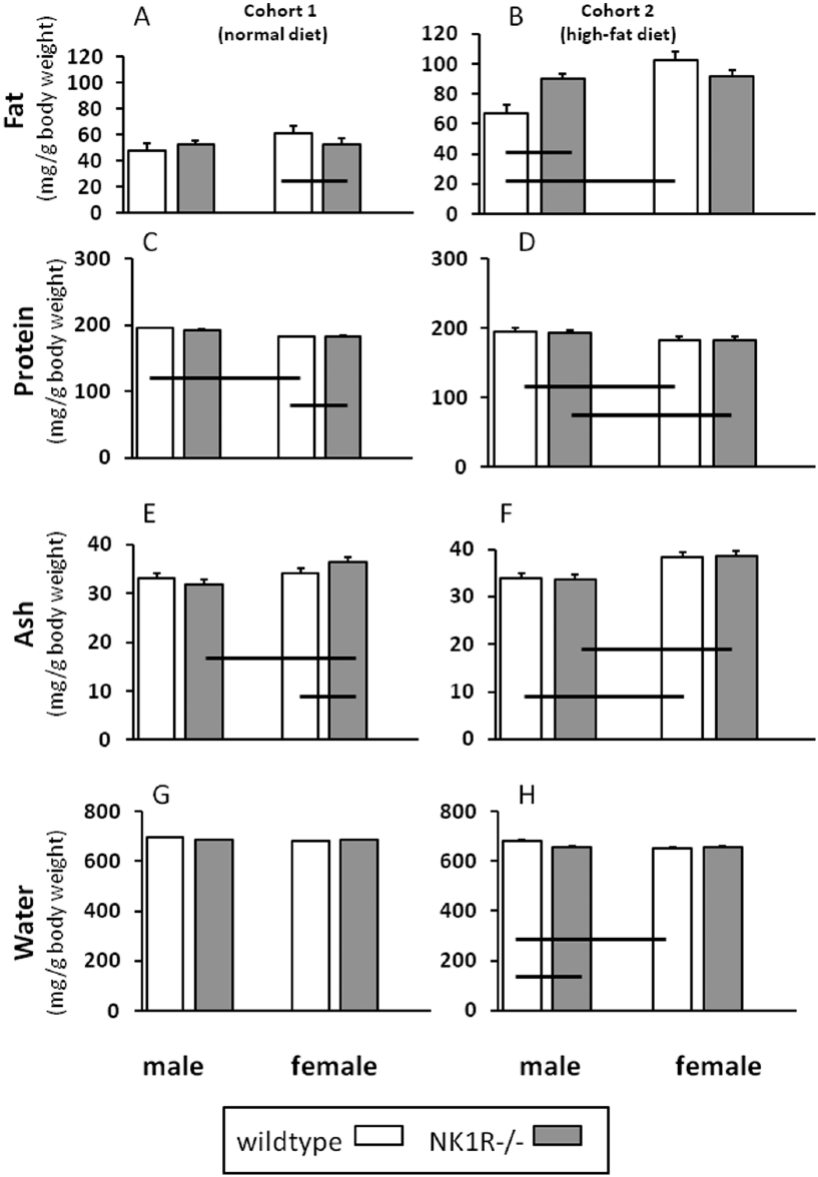
Body composition (mg/g body weight) of NK1R-/- mice and wildtype male and female mice fed either standard laboratory chow (Cohort 1) or a high-fat (‘Western’) diet (Cohort 2). *N* = 8–10 per group. Data show mean ± SEM. Solid lines, linking two groups indicate statistically significant differences of at least *p* < 0.05. (A, B) Fat; (C, D) protein; (E, F) ash; (G, H) water. The fat content of male NK1R-/- mice from Cohort 2 (given the high-fat diet) was higher than that of male wildtypes but did not differ from females of either genotype.

In Cohort 1, there was no overall genotype difference in fat/g body weight. However, female, but not male, NK1R-/- mice had slightly less fat than their wildtypes ((–9%): geno*sex: F_(1,35)_ = 7.03, *p* < 0.05; *post-hoc* LSD: *p* = 0.03) (Figure 3(A)).

In the batch of animals fed the high-fat diet (Cohort 2), it is striking that the fat content of male NK1R-/- mice was considerably higher than their male wildtypes ((+35%); geno*sex: F_(1,33)_ = 10.44, *p* < 0.01). Moreover, the fat content of male wildtypes was less than that of female wildtypes (sex: F_(1,33)_ =12.22, *p* < 0.001; *post-hoc* LSD *p* < 0.0001], but there was no difference in the fat content of the two genotypes of female mice (Figure 3(B)).

### Protein (mg/g body weight)

There was no difference in the amount of protein in the two genotypes from either cohort. However, there was an overall difference between the two sexes ((RANK transformed data): F_(1,36)_ = 8.56, *p* < 0.01 (Cohort 1); (raw data): F_(1,33)_ = 37.71, *p* < 0.01 (Cohort 2)) with females typically having slightly less protein (ca. 3%) than males (Figure 3(C, D)).

### Ash (mg/g body weight)

There was no difference in the ash content of the two genotypes in either cohort (Figure 3(E and F)). However, female mice had a higher ash content than their males in both cohorts (F_(1,36)_ = 15.39, *p* = 0.001 (Cohort 1); F_(1,36)_ = 39.5, *p* = 0.001 (Cohort 2)), especially in the NK1R-/- genotype (LSD: *p* < 0.001).

### Water (mg/g body weight)

Neither genotype nor sex affected water content of mice from Cohort 1 (Figure 3(G)), but an overall difference between the two genotypes in Cohort 2 was of borderline significance (F_(1,33)_ = 3.57, *p* = 0.067). Furthermore, in the latter cohort, there was a difference between the two sexes (F_(1,33)_ = 9.12, *p* = 0.005) and an interaction between genotype and sex (F_(1,33)_ = 10.31, *p* < 0.01) such that male wildtype mice had a higher water content than both male NK1R-/- mice (*p* < 0.01) and female wildtypes (*p* < 0.001) but the differences were small (ca. 4% and 9.5%, respectively) (Figure 3(H)).

## Discussion

The neurochemical and behavioural abnormalities expressed by NK1R-/- mice have led us to propose that a functional deficit of NK1R could contribute to ADHD in patients with polymorphism of the *TACR1* gene (the human equivalent of *Nk1r* in rodents) (e.g. Yan et al., 2011; see also Sharp et al., 2014). Prompted by reports of associations between both small body size and obesity with ADHD, we have investigated whether there are differences in the body size, body mass and/or composition of NK1R-/- mice and their wildtypes that would be consistent with their status as a murine analogue of humans with ADHD. This study has established that these physical characteristics are abnormal in NK1R-/- mice and so, in combination with impaired cognitive performance, these factors could serve as biomarkers in translational studies to help to distinguish ADHD patients with *TACR1* polymorphism(s) from those with ADHD of different aetiology.

The first experiment compared the physical characteristics of NK1R-/- and wildtype mice that had been weaned onto normal lab chow. Both male and female NK1R-/- mice were approximately 7% shorter than their wildtypes: the genotype difference in male mice was confirmed in the second cohort. This finding echoes reports of an association between small body size and ADHD (Hanć et al., 2015; Spencer et al., 1996). Hitherto, the role of NK1R or substance P on gross physical development has not been investigated, but our finding suggests that *TACR1* dysfunction could contribute to small body stature in some ADHD patients.

Consistent with their small body size, the weight of male NK1R-/- mice fed a normal diet was lower (ca.10%) than their wildtypes and yet their mBMI did not differ. Moreover, both the weight of female NK1R-/- mice and their mBMI body density was higher (ca.12%) than their wildtypes. These findings suggest that body mass is disproportionally higher than normal in mice that lack functional NK1R. This would explain why the tendency for small body size of ADHD patients is not necessarily paralleled by a lower body weight (Hanć et al., 2015; Spencer et al., 1996).

In Cohort 2, we investigated whether this physical profile was also evident when the mice were fed a Western (high-fat) diet. Although NK1R-/- males were again shorter than their wildtypes, the body weight of the two genotypes of male mice did not differ, as was also the case for females. This is reflected by the higher mBMI of both male and female NK1R-/- mice, compared with their wildtypes.

After correction for body weight, the amount of food consumed by the two genotypes was similar, across all test groups. This means that we cannot distinguish whether there is a primary increase in food intake by NK1R-/- mice that drives an increase in body mass and mBMI, or whether there is a primary metabolic disturbance that accounts for both the increase in body mass, mBMI and the increase in food intake. Although we cannot rule out the former, the latter is more likely in view of the striking difference in fat content of NK1R-/- and wildtype mice. Whereas the total fat content of female NK1R-/- mice from Cohort 1 was more than 17% lower than that of their wildtypes, there was no such difference in Cohort 2, which was fed the high-fat diet. Furthermore, there was no appreciable difference in the fat content of males from Cohort 1 and yet the total fat content of male NK1R-/- mice from Cohort 2 was 35% higher than that of their wildtypes. It seems that, when fed a high-fat diet, fat accumulates in both genotypes, but NK1R-/- mice are more prone to fat accumulation than wildtypes: especially the males. Nevertheless, we acknowledge that a head-to-head comparison of the two sexes, genotypes and diets is needed to confirm this inference.

These differences in fat content of NK1R-/- and wildtype mice contrast strikingly with the lack of any appreciable genotype differences in protein, water or ash in either males or females. Ash comprises the non-combustible material in the carcass and is mainly, but not exclusively, derived from bone. As a consequence, ash content offers only an approximate index of bone mass. Moreover, it was not feasible to measure bone volume in combination with the techniques used here and so we were unable to ascertain whether there were any genotype and / or diet induced differences in bone density. As a consequence, despite females typically producing more ash than males, we cannot infer that this is paralleled by a higher bone mineral density but this possibility should be considered in any future translational studies.

The smaller size of NK1R-/- mice on the normal diet would explain why they weighed less than the wildtypes, despite their higher mBMI. It has been suggested that the association between obesity and ADHD is a secondary consequence of features of the disorder that will tend to promote food intake and weight gain: such as impulsive eating, disruption of feeding architecture or impaired neuronal reward circuits (reviewed by Martínez de Velasco et al., 2015). It has also been reported that being small for gestational age increases the risk of obesity (Grissom and Reyes, 2013) and that maternal adiposity and obesity increase risk of inattention (Rodriguez, 2010) and ADHD in the offspring (Chen et al., 2014; reviewed by Rivera et al., 2015). Obviously, it is hard to distinguish the direction of risk causation in these human studies. Nevertheless, it would be interesting to carry out a head-to-head comparison of the effect of diet on the performance of the two genotypes in the 5-CRSTT to test the possibility that a high-fat diet aggravates ADHD, as has been suggested for humans (Howard et al. 2011), for which there is some preclinical supporting evidence (Marwitz et al., 2015). A further interesting caveat is that circadian rhythms are disrupted in these mice (Porter et al., 2015b) and that disruption of circadian rhythms can increase vulnerability to both obesity and ADHD (Vogel et al., 2015).

Our findings are consistent with evidence that activation of the substance P/NK1R system reduces fat storage by adipocytes *in vitro* (Miegueu et al., 2013) and that substance P promotes lipolysis, even if only indirectly (see Ng, 1990). If this is the case, it could also help to explain the association between obesity and ADHD (Erhart et al., 2012; Pagoto et al., 2009; Waring and Lapane, 2008), even in adults (Cortese et al., 2016). By contrast, there are reports that administration of the NK1R antagonist,CJ12255, prevents weight gain and fat accumulation in male mice given a high-fat diet and that of obese *ob/ob* mice (Karagiannides et al., 2008) or allergen-sensitized obese mice (Ramalho et al., 2013). However, this drug also reduced food intake, which was not the case for NK1R-/- mice in this study, and so a reduction in body weight and adiposity could reflect a non-specific response. For example, many NK1R antagonists also block L-type Ca^2+^ channels (see Stanford, 2014): this leads to a reduction in body weight through augmentation of thermogenesis, albeit indirectly (Zhao et al., 1999).

The sex differences in both mBMI and fat content on the high-fat diet are interesting, not least because there are reports that an interaction between activation of NK1Rs and sex could influence adiposity. For instance, NK1R-mediated stress responses are influenced by oestradiol (Bradesi et al., 2003) and there is evidence for sexual dimorphism of NK1R expression in many tissues (Villablanca and Hanley, 1997). Moreover, a recent PET study using the highly selective NK1R [*TACR1*] ligand, [^11^C]GR205171, found a lower density of NK1Rs in the thalamus of women (Engman et al., 2012).

In summary, our findings refine the profile of the NK1R-/- mouse phenotype. They indicate that female NK1R-/- mice have higher mBMI than wildtypes, regardless of diet, and that a lack of functional NK1R in males increases their risk of an excessive increase in mBMI and body fat when fed a high-fat (Western) diet. These findings highlight the need to incorporate the quality of the diet as an experimental variable when comparing genotype differences in body mass and composition. With that proviso, the findings are consistent with reports of a tendency for unmedicated ADHD patients to have higher percentage body fat and shorter body stature than other subjects. We have yet to study the cognitive performance and response control of female NK1R-/- mice and so do not know whether they are abnormal. However, one study does suggest that the females, unlike males, are not hyperactive (Porter et al., 2015a). Nevertheless, our findings lead to the prediction that a subset of ADHD patients with *TACR1* polymorphism(s), especially males, would tend to have short stature, compared with other subjects, and that the males would have an increased risk of developing obesity when fed a Western diet.
